# Physical Diagnosis in Obstetrics

**Published:** 1902-12

**Authors:** 


Physical Diagnosis in Obstetrics. A guide in ante-partum, partum and post-partum examinations. For the use of physicians and undergraduates. By Edward A. Ayres, M. D., Professor of Obstetrics in the New York Polyclinic, etc. With Illustrations. Pages, 283; price, $2.00. New York: E. B. Treat & Co., 241-243 West 23rd St. 1901.

A study of this volume systematizes and brightens the physicians' diagnostic ability in obstetric practice. The author's many years of clinical instruction has made him high authority in the branch he teaches, and the student in obstetrics will find in the present work a concise and practical treatise. A valuable chart is suggested and includes a view of wide range, including ante-partum examination, uterometry, pelvimetry and abdominal palpation, vaginal examination, history of labor, breasts, child's history, maternal past-partum history, final examination and a summary of the prominent features of the case. T. J. B.

